# Mediating effect of psychological distress and mindful eating behaviors between orthorexia nervosa and academic self-efficacy among Lebanese university female students

**DOI:** 10.1186/s12889-024-17812-7

**Published:** 2024-02-02

**Authors:** Muna Barakat, Nesreen A Salim, Diana Malaeb, Mariam Dabbous, Fouad Sakr, Souheil Hallit, Feten Fekih-Romdhame, Sahar Obeid

**Affiliations:** 1 Department of Clinical Pharmacy and Therapeutics, Faculty of Pharmacy, Applied Science Private University, Amman, Jordan; 2 Middle East University Research Unit, Middle East University, Amman, Jordan; 3 Prosthodontic Department, School of Dentistry, The University of Jordan, Amman, Jordan; 4 Prosthodontic Department, Jordan University Hospital, Amman, Jordan; 5 College of Pharmacy, Gulf Medical University, Ajman, United Arab Emirates; 6 School of Pharmacy, Lebanese International University, Beirut, Lebanon; 7 School of Medicine and Medical Sciences, Holy Spirit University of Kaslik, Jounieh, P.O. Box 446, Lebanon; 8 The Tunisian Center of Early Intervention in Psychosis, Department of Psychiatry “Ibn Omrane”, Razi hospital, 2010 Manouba, Tunisia; 9 Faculty of Medicine of Tunis, Tunis El Manar University, Tunis, Tunisia; 10 School of Arts and Sciences, Social and Education Sciences Department, Lebanese American University, Jbeil, Lebanon

**Keywords:** Academic performance, Orthorexia nervosa, Mindful eating, Psychological distress

## Abstract

**Objectives:**

This study examined the mediating effect of psychological distress and mindful eating behaviors between orthorexia nervosa and academic self-efficacy among Lebanese university female students.

**Methods:**

A total of 769 female participants enrolled in this cross-sectional study (mean age 21.58 ± 3.20 years). A self-administered questionnaire was distributed among university female students. The questionnaire consisted of Mindful Eating Behaviors Scale, ORTO-R, Depression Anxiety Stress Scale, and Arabic version of Academic Self-Efficacy Scale.

**Results:**

The results showed that psychological distress fully mediated the association between orthorexia nervosa and academic self-efficacy; higher orthorexia nervosa was significantly associated with less psychological distress (β= -0.31, *p* =.05), with more psychological distress significantly associated with lower academic self-efficacy (β= -0.32, *p* =.09). Focused eating fully mediated the association between orthorexia nervosa and academic self-efficacy; higher orthorexia nervosa was significantly associated with less focused eating (β=-0.09, *p* =.04), with more focused eating significantly associated with better academic self-efficacy (β = 1.40, *p* =.10). Orthorexia nervosa was not directly associated with academic self-efficacy in both models.

**Conclusion:**

This study shed light on important connections between orthorexia nervosa, psychological distress, mindful eating behaviors, and academic self-efficacy within the Lebanese context. The findings will have practical implications for both educational institutions and healthcare providers striving to support young female adults’ overall well-being and academic success.

## Introduction

Academic self-efficacy is the term used to describe students’ attitudes and ideas about their potential for academic success [1]. It also includes their confidence in completing assignments and understanding the subject well [1]. Academic self-efficacy can act as a direct or indirect indicator of academic achievement and performance [2, 3]. Previous studies indicated that students’ academic self-efficacy and performance are affected by many factors (self-motivation, family income, life perspective, student background), which carry substantial influence [4, 5]. Students perceive that factors associated with the institution significantly impact their performance, primarily because they prefer a serene and conducive university environment [4, 5]. Additionally, students hold the belief that educators with effective teaching skills and a diverse range of teaching methods positively influence their academic outcomes [6, 7]. In recent years, researchers have focused on exploring the relationships between academic performance and several key indicators related to physical and mental health [2, 8]. Among the key mental health correlates of academic self-efficacy and performance is eating disorders [9–11].

### The relationship between orthorexia nervosa and academic self-efficacy

**Fig. 1:**
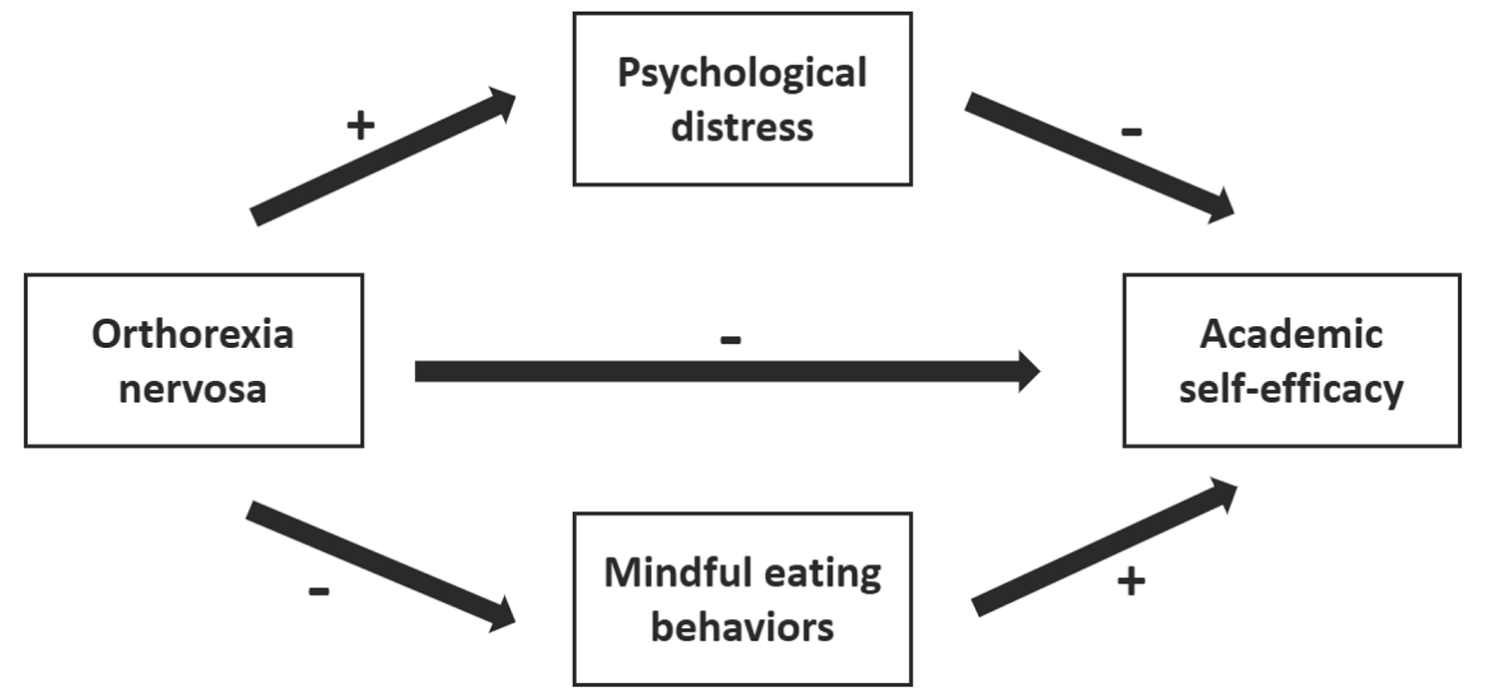
A conceptual framework illustrating the pathways between study variables

In various scientific domains, there has been a notable surge in interest regarding health and the adoption of wholesome eating habits [12–17]. Our dietary choices impact not only physical development and growth but also overall fitness and well-being [13, 15, 16]. However, an excessive fixation on the quality of food can potentially have negative consequences. The phenomenon of Orthorexia Nervosa (ON) has garnered considerable attention in recent research. ON involves an excessive preoccupation with food quality, meticulous food preparation, and stringent nutritional criteria [18]. While it’s not officially recognized as a distinct eating disorder in widely accepted diagnostic manuals like the Diagnostic and Statistical Manual of Mental Disorders (DSM-5), it has been discussed in the literature as a concept related to disordered eating [18]. ON is different from anorexia nervosa, which is typically focus on the quantity of food and the fear of gaining weight and well defined in DSM-5 [19]. People with anorexia often have a distorted body image and engage in extreme efforts to restrict their calorie intake, leading to significant weight loss [19]. Both orthorexia and clinical eating disorders exist on a spectrum of disordered eating behaviors [20]. There may be some overlap between orthorexia and other eating disorders, but they are distinct in their primary focus and motivation [20]. In parallel, research has shown that individuals with ON may experience adverse effects on their academic performance due to rigid dietary restrictions that can lead to nutrient deficiencies and impaired cognitive functioning [21]. However, there is to date scarce research focusing on the relationship between ON and academic outcomes. The present study proposes to add to the body of knowledge available by examining the role of possible mediators (i.e., mindful eating and psychological distress) in this relationship. Figure 1 illustrates the proposed conceptual framework representing the expected relationships between this study’s variables.

### Mindful eating and psychological distress as possible mediators

Mindful eating is the act of eating while being in a state of non-judgmental awareness, shifting one’s attention to the food and mind-body connection. Thus, allowing exploration of the complex cognitive-biological experience of eating [22, 23]. This healing eating mode favorably affects problematic eating habits (e.g., desensitizing hunger and satisfaction cues) and digestive disturbances attributed to stress [22, 23]. Mindfulness has been demonstrated to have a negative association with various aspects of disordered eating, including dietary restrictions and obsessive preoccupations with food [24]. These behaviors are often observed in ON-individuals, emphasizing the potential relevance of mindfulness in addressing ON [24]. Additionally, Strahler’s research indicates that mindfulness can moderate orthorexia eating tendencies while also offering protective effects against eating disorder pathology [25]. As a result, although research on this topic is limited, it hints at the direct influence of mindfulness on ON. This lays the groundwork for further investigation in this area, particularly focusing on mindfulness practices specific to eating behaviors. Additionally, mindful eating behaviors have positively influenced academic outcomes [5]. By practicing awareness and being present while consuming food choices that nourish both body and mind, students may experience increased concentration levels that translate into improved academic outcomes [26].

Psychological distress is another significant aspect affecting academic outcomes [27]. Students facing stressors such as anxiety or depression may struggle with maintaining focus and motivation, leading to decreased productivity in their studies [28]. It is worth noting that prior research has examined the role of psychological distress and mindful eating as potential mediators between eating disorders and various factors, but notably, academic self-efficacy was not included in these studies [13, 29].

### The present study

In this study, concentrating on female students aimed to provide a more targeted exploration of the pathways between ON, psychological distress, mindful eating and academic self-efficacy within a specific demographic, as existing literature suggests that ON and its associations with psychological distress and mindful eating behaviors may exhibit gender-specific patterns (e.g., [30, 31]). Understanding these factors can have significant implications for educational institutions and healthcare providers in developing appropriate interventions or support systems for students struggling with orthorexia nervosa or related issues that may impact their academic self-efficacy. Furthermore, conducting this study in Lebanon allows us to contribute to the limited existing research on orthorexia nervosa within Middle Eastern countries. Therefore, this study aimed to examine the mediating effect of psychological distress and mindful eating behaviors between ON and academic self-efficacy among a sample of Lebanese university female students.

## Methods

### Study design

A self-administered anonymous questionnaire utilizing Google Forms was used for a cross-sectional study conducted between May and August 2023. Using the snowball method, the link was distributed among the female university students, who fulfilled the following inclusion criteria: (1) 18 years or older and (2) Lebanese student. Excluded were those who refused to participate in the study. The research team approached students, who were asked to forward the link to other friends. After providing digital informed consent, participants were asked to complete the questionnaire, which was presented in a pre-randomised order to control for order effects. The survey was anonymous and participants completed the survey voluntarily and without remuneration, in approximately 20 min on average.

### Minimal sample size

A minimal sample of 410 was deemed necessary using the formula suggested by Fritz and MacKinnon [32] to estimate the sample size: $n=\frac{L}{f2}+k+1$, where f=0.14 for small effect size, L=7.85 for an α error of 5% and power β = 80%, and k=8 variables to be entered in the model.

### Questionnaire

The questionnaire consisted of two main sections. The first section was sociodemographic, including age (in years), Household Crowding Index (number of persons divided by number of rooms in the house) [33], Financial burden (self-reported on a scale from 1 to 10, with 10 indicating heaviest burden), Body Mass Index (calculated according to the World Health Organization formula [34]), Physical activity index (calculated by multiplying the intensity by the strength by the frequency of the physical activity [35]). We also asked questions related to the student specialization (Scientific/Non-scientific), alcohol drinking status, regularity of menses status, presence of pelvic pain, and cigarette and waterpipe smoking status.

The second section consisted of the following scales:

*Mindful Eating Behaviors Scale* [36]. Validated in Lebanon [37], it is composed of 17 items rated on a 5-point Likert scale (1 = never to 5 = very often). This scale yields four different domains of attentive mindful eating: Focused Eating (5 items), Eating in response to Hunger and Satiety Cues (5 items), Eating with Awareness (3 items), and Eating Without Distraction (4 items). Higher scores reflect more mindful eating behaviors. The results of the reliability analysis of the four domains showed the following results: Focused Eating (ω = 0.93/ α = 0.93), Eating in response to Hunger and Satiety Cues (ω = 0.92/ α = 0.92), Eating with Awareness (ω = 0.85/ α = 0.84), and Eating Without Distraction (ω = 0.81/ α = 0.81).

*ORTO-R*. Validated in Lebanon [38, 39], the ORTO-R is the revised version of the ORTO-15 [40] used to assess orthorexia nervosa tendencies. ORTO-R has been suggested due to the unstable factor structure of the ORTO-15 across multiple populations. It is a six-item scale [41], scored from 0 (never) to 3 (always). Those 6 items were identified as the best markers of orthorexia nervosa. More orthorexia nervosa traits and behaviors are indicated by higher ORTO-R scores [42] (ω = 0.80/ α = 0.80).

*Depression Anxiety Stress Scale (DASS-8).* Validated in Arabic [43], it’s a self-report questionnaire used to measure psychological distress. This scale is composed of eight items, in three subscales: depression (three items including ‘’ felt down hearted and blue’’), anxiety (three items including: ‘’ felt scared without reason’’), stress (two items including: ‘’ was using a lot of my mental energy ‘’), rated on a 4-point Likert scale: ranging from ‘’0 = Did not apply to me at all’’ to ‘’3 = Applied to me very much or most of the time’’, higher scores indicate more psychological distress [43] (ω = 0.92/ α = 0.92).

*Arabic version of the Academic Self-Efficacy Scale (ASE)* [44] is an eight-item rapid assessment instrument developed by Chemers et al. (2001) [45] for the purposes of measuring a student’s confidence in their ability to successfully perform several academic tasks. Each item is scored on a 7-point Likert scale (1 = strongly disagree to 7 = strongly agree) (ω = 0.97/ α = 0.97).

### Statistical analysis

The SPSS software v.26 was used for the statistical analysis. No missing data was found in our database as all questions were required in the Google form. McDonald’s ω and Cronbach’s α were computed for reliability analysis. The academic self-efficacy score was considered normally distributed since the skewness and kurtosis values varied between ± 1.96. The Student *t* test was used to compare two means and the Pearson test to correlate two continuous variables. The mediation analysis was conducted using PROCESS MACRO (an SPSS add-on) v3.4 model 4 [46]; four pathways derived from this analysis: pathway A from the independent variable to the mediator, pathway B from the mediator to the dependent variable, Pathways C and C’ indicating the total and direct effects from the independent to the dependent variable. Results were adjusted over all variables that showed a *p* <.25 in the bivariate analysis. We considered the mediation analysis to be significant if the Boot Confidence Interval did not pass by zero. *P* <.05 was deemed statistically significant.

## Results

A total of 769 participants enrolled in this study (mean age = 21.58 ± 3.20 years). Other characteristics of the sample can be found in Table 1.

**Table 1:** Sociodemographic and other characteristics of the participants (*n* = 769)

Variable	n (%)
**Specialization**	
Scientific	602 (78.3%)
Non-scientific	167 (21.7%)
**Alcohol drinking**	
No	689 (89.6%)
Yes	80 (10.4%)
**Regular menses**	
No	168 (21.8%)
Yes	601 (78.2%)
**Pelvic pain**	
No	597 (77.6%)
Yes	172 (22.4%)
**Cigarette smoking**	
No	720 (93.6%)
Yes	49 (6.4%)
**Waterpipe smoking**	
No	604 (78.5%)
Yes	165 (21.5%)
	**Mean ± SD**
Age (years)	21.58 ± 3.20
Household crowding index	1.28 ± 0.58
Financial burden	5.30 ± 2.59
Body Mass Index (kg/m^2^)	22.61 ± 3.75
Physical activity index	22.28 ± 18.00
Psychological distress	10.75 ± 6.68
Hunger and satiety cues	12.01 ± 5.25
Eating with awareness	4.35 ± 3.23
Eating without distraction	6.34 ± 3.89
Dysmenorrhea	6.05 ± 2.45

### Bivariate analysis

The bivariate analysis results are shown in Tables 2 and 3. A higher academic self-efficacy score was significantly found in participants who have a scientific major, who do not have pelvic pain and who do not smoke cigarettes and waterpipe. Moreover, higher academic self-efficacy was significantly correlated with more focused eating and hunger and satiety cues, whereas more psychological distress was significantly associated with lower academic self-efficacy.

**Table 2:** Bivariate analysis of the categorical variables associated with academic self-efficacy

Variable	Mean ± SD	T	df	*p*
**Major**		2.40	767	**0.017**
Scientific	41.00 ± 15.20			
Non-scientific	37.77 ± 15.94			
**Alcohol drinking**		− 0.63	767	0.529
No	40.18 ± 15.32			
Yes	41.33 ± 16.18			
**Cigarette smoking**		3.49	767	**0.001**
No	40.80 ± 15.29			
Yes	32.92 ± 15.38			
**Waterpipe smoking**		1.98	767	**0.049**
No	40.87 ± 15.38			
Yes	38.20 ± 15.39			

Numbers in bold indicate significant *p* values

**Table 3:** Correlation matrix of continuous variables

Variable	1	2	3	4	5	6	7	8	9	10	11
1. Academic self-efficacy	1										
2. Age	0.001	1									
3. Household crowding index	0.02	-0.06	1								
4. Financial burden	-0.03	-0.03	0.17***	1							
5. Body Mass Index	-0.01	0.16***	-0.04	0.10**	1						
6. Physical activity index	0.06	0.02	0.02	-0.06	0.06	1					
7. Psychological distress	-0.14***	0.003	0.03	0.22***	0.13***	-0.03	1				
8. Focused eating	0.47***	0.01	0.06	0.01	-0.01	0.08*	-0.03	1			
9. Hunger and satiety cues	0.41***	0.04	0.03	-0.07	-0.13***	0.06	-0.13***	0.71***	1		
10. Eating with awareness	-0.07	-0.04	-0.01	0.04	0.12**	0.02	0.18***	0.05	0.12**	1	
11. Eating without distraction	0.01	-0.06	-0.03	0.06	0.07*	-0.002	0.21***	0.13***	0.19***	0.65***	1

Numbers in the table reflect Pearson correlation coefficients; *p <.05; **p <.01; ***p <.001

### Mediation analysis

**Fig. 2:**
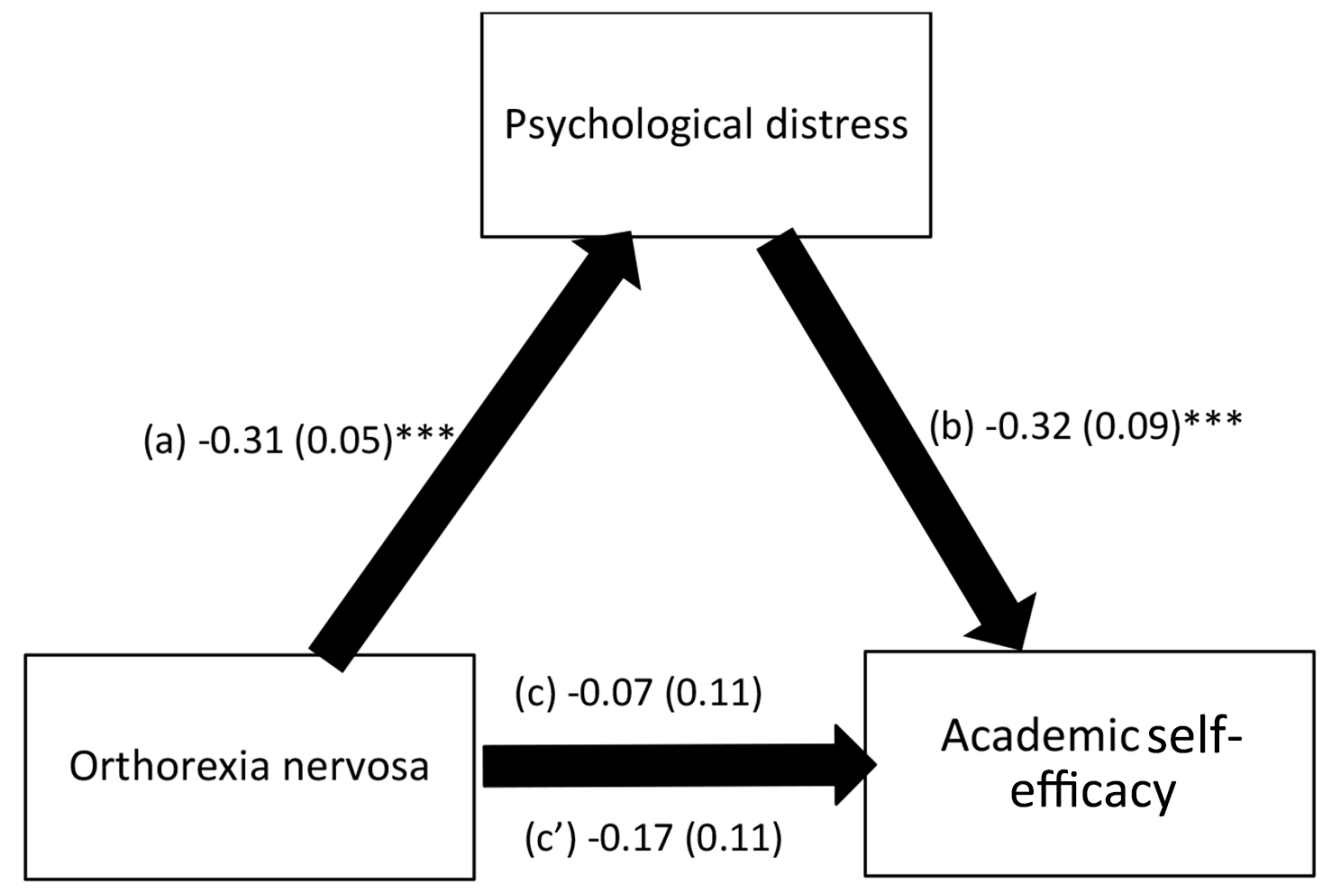
**(a)** Relation between orthorexia nervosa and psychological distress (R^2^ =.084); **(b)** Relation between psychological distress and academic self-efficacy (R^2^ =.046); **(c)** Total effect of orthorexia nervosa on academic self-efficacy (R^2^ =.052); (c’) Direct effect of orthorexia nervosa on academic self-efficacy. Numbers are displayed as regression coefficients (standard error). ***p <.001

The results of the mediation analysis are summarized in Table 4. Results were adjusted over the following variables: major, smoking cigarettes, smoking waterpipe and physical activity index. The results showed that psychological distress fully mediated the association between orthorexia nervosa and academic self-efficacy; higher orthorexia nervosa was significantly associated with less psychological distress, with more psychological distress significantly associated with lower academic self-efficacy (Fig. 2).

**Fig. 3:**
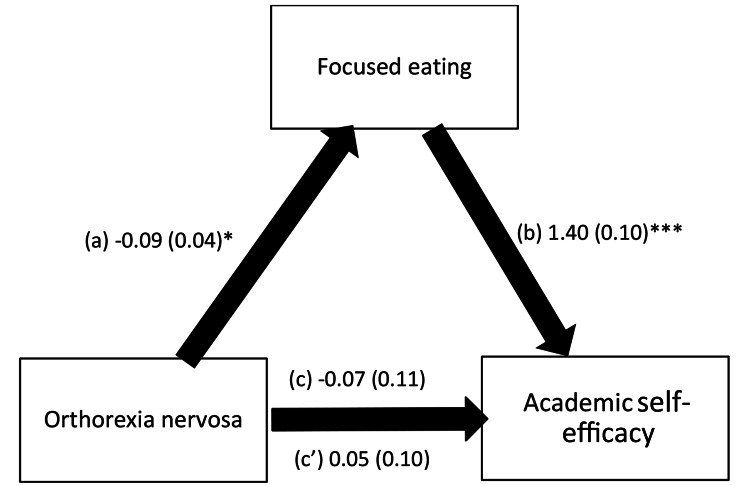
**(a)** Relation between orthorexia nervosa and focused eating (R^2^ =.031); **(b)** Relation between focused eating and academic self-efficacy (R^2^ =.233); **(c)** Total effect of orthorexia nervosa on academic self-efficacy (R^2^ =.052); (c’) Direct effect of orthorexia nervosa on academic self-efficacy. Numbers are displayed as regression coefficients (standard error). * *p* <.05; ***p <.001

Focused eating fully mediated the association between orthorexia nervosa and academic self-efficacy; higher orthorexia nervosa was significantly associated with less focused eating, with more focused eating significantly associated with better academic self-efficacy (Fig. 3). It is noteworthy that orthorexia nervosa was not directly associated with academic self-efficacy in both models.

**Table 4:** Mediation analyses results, taking orthorexia nervosa as the independent variable, psychological distress/ mindful eating behaviors subscales as mediators and academic self-efficacy as the dependent variable

	Direct effect			Indirect effect		
Mediators	Beta	SE	*P*	Beta	Boot SE	Boot CI
Psychological distress	-0.17	0.11	0.123	0.10	0.03	0.04; 0.17*
Focused eating	0.05	0.10	0.633	-0.12	0.05	-0.23; -0.02*
Hunger and satiety cues	-0.05	0.10	0.626	-0.02	0.05	-0.12; 0.07
Eating with awareness	-0.10	0.11	0.354	0.03	0.02	-0.003; 0.07
Eating without distraction	-0.07	0.11	0.546	-0.01	0.02	-0.05; 0.04

*indicates significant mediation. Direct effect refers to the direct association between orthorexia nervosa and academic self-efficacy without the effect of the mediator, whereas the indirect effect refers to the same association through the mediator (psychological distress/mindful eating behaviors)

## Discussion

### ON, psychological distress and academic self-efficacy

The current study demonstrated that higher ON was significantly associated with less psychological distress. This finding resonates with the results of previous study that examined Lebanese participants who met the ON criteria who experienced decreased psychological distress [15]. A recent study found that ON was only marginally connected with psychological disorders such as depression, anxiety, and stress, and this effect was reduced in a German sample by healthy orthorexia [47]. Additionally, scientific studies have established a longstanding connection between nourishing diets—especially those abundant in fruits, vegetables, fish, and whole grains—and a reduced likelihood of experiencing depression [12]. On the other hand, roughly 50% of individuals who adopt restrictive eating behaviors fulfill the requirements for a diagnosis of Generalized Anxiety Disorder (GAD). Additionally, about 15% of those grappling with eating disorders also experience social phobia, as indicated by research [48]. Conditions like major depressive disorder and various anxiety disorders, including GAD, have been identified as potential predisposing factors for the development of ON) [16]. These diverse findings could be attributed to many factors, a specific degree of stress has the potential to lead to adaptive responses, such as the development of a health-conscious focus on diet, known as healthy orthorexia. Stress exists on a spectrum of varying intensities, each prompting behaviors that can either be beneficial or detrimental [49].

The negative correlation of psychological elements, like anxiety and stress and ON in this study, implies the possibility of other factors influencing the connection between experiencing an eating disorder (ON) or following a typical pattern (healthy orthorexia) [29]. It is worth noting that anxiety is present in both individuals with maladaptive eating patterns like ON and those with regular eating patterns like healthy orthorexia, albeit with varying degrees of intensity [13]. Moreover, discrepant results could be explained using different measuring instruments, different demographic characteristics and cultures [14, 17].

Another important finding was that psychological distress was significantly associated with lower academic self-efficacy. Consistent with our findings, several recent studies indicated a robust connection between indications of psychological discomfort, one’s belief in their academic capabilities, and their advancement in studies [11, 50–52]. Students who communicated experiencing intense psychological distress were four times more prone to indicating low confidence in their academic abilities and twice as prone to noting delayed advancement in their studies, in contrast to students who disclosed mild or moderate mental distress symptoms. Among those who conveyed severe mental distress, 27% had actively sought professional assistance, while 31% had contemplated seeking help [51]. A comprehensive review, encompassing 11 studies across three distinct countries, substantiated a definite correlation between subpar emotional well-being and a setback in academic advancement [53]. Depressive disorders lead to decreased mood, impaired cognitive abilities, a diminished sense of coping and interpersonal interest, alongside decreased energy levels. In a reciprocal manner, both depression and anxiety frequently impact memory and concentration, thereby complicating the process of acquiring fresh knowledge and effectively dealing with examination scenarios [54, 55].

Distress, academic self-efficacy, and ON are interconnected psychological variables that can influence and reinforce each other. The mediating effect of psychological distress suggests that it could act as an intermediary factor between ON and academic self-efficacy. Comprehending this mediating role can be highly beneficial for designing prevention and intervention approaches. Collaborative efforts between educational institutions and mental health professionals can be employed to offer assistance and resources to students grappling with academic stress [11, 56, 57]. This, in turn, can help mitigate the likelihood of resorting to unhealthy coping mechanisms such as Orthorexia Nervosa. The mediating impact of psychological distress in the association between academic self-efficacy and Orthorexia Nervosa underscores the significance of addressing mental health issues within educational settings [20]. It underscores that academic achievement and mental well-being are interconnected and should be holistically considered to foster positive behaviors and outcomes.

### ON, mindful eating and academic self-efficacy

The aspect of “focused eating” within the mindful eating behavior subscale displayed a significant negative relationship with ON (higher orthorexia nervosa was significantly associated with less focused eating). Although this association was proved in previous studies in opposite direction to this study (the influence of mindful eating on ON was the tested association), research findings have consistently indicated an inverse connection between trait mindfulness and the symptoms of disordered eating [58, 59]. Absolutely, promoting mindfulness in the context of eating can indeed have a positive impact on addressing problematic eating behaviors [60]. Certainly, mindful eating has been linked to a decrease in symptoms of eating disorders and restrictive eating [1, 38]. In a study by Taylor, Daiss, and Krietsch (2015), it was discovered that individuals with higher scores in mindful eating exhibited lower levels of eating disorders symptoms, particularly those related to constant thinking about food. This preoccupation with food is a fundamental aspect of ON [40]. As such, it is plausible to anticipate a similar positive influence within the context of ON. Furthermore, there is accumulating evidence that interventions grounded in mindfulness are proving to be effective treatments for eating disorders [61]. Additionally, focused eating was significantly associated with better academic self-efficacy in this study. Previous studies indicated that healthy eating habits have a positive effect on students’ academic performance [62–64]. In times of academic stress, individuals may resort to focused eating as means of coping. The practice of carefully organizing and regulating one’s dietary choices can create a feeling of structure and accomplishment, offering a temporary relief from the pressures associated with academic self-efficacy and improve academic performance. Accordingly, focused eating was a mediator between the academic self-efficacy and ON with no direct association between academic self-efficacy and ON. In this context, the term “mediator” signifies that focused eating might function as an intermediate factor that sheds light on the connection between academic self-efficacy and ON. It implies that individuals with strong academic self-efficacy may be more inclined to practice focused eating, which, in turn, could lower the risk of developing ON by adopting a healthy lifestyle. It is crucial to emphasize that ON will not necessarily develop in all individuals with low academic self-efficacy, and not everyone who adopts focused eating habits will necessarily excel academically. The association between these factors can greatly vary among individuals and is influenced by various personal, cultural, and environmental factors.

The findings suggest a potential link between academic self-efficacy, focused eating, and ON, with focused eating potentially playing a mediating role by acting as a coping mechanism for academic stress. However, it is essential to acknowledge that individual experiences and outcomes can diverge significantly, and further research is required to fully comprehend the intricate interplay among these variables.

### Clinical implications

Our study sheds light on mindful eating behaviors, academic self-efficacy, and orthorexic eating, along with their connections to sociodemographic factors and mediating relationships. Given that orthorexia is a relatively new eating disorder not yet fully recognized as a distinct pathology, our findings pave the way for further exploration of ON, both within Lebanon and on a global scale. At institutional level, school-based mental health professionals can benefit from our findings by tailoring interventions to address orthorexia nervosa among students, and help mitigate its possible negative effects on their academic self-efficacy. The identification of mediating relationships underscores the importance of comprehensive assessments that consider both psychological distress and mindful eating practices. This knowledge can inform targeted interventions to promote healthy eating habits and psychological well-being among university students. To this end, school-based interventions integrating mindfulness, which showed promising results in improving academic outcomes [56], can be implemented. There remains substantial groundwork to be covered in terms of enhancing the utilization and acceptability of mindful eating interventions for effectively altering eating behaviors. Future research is warranted and should explore the development and implementation of evidence-based interventions targeting orthorexia nervosa, mindful eating behaviors, and academic self-efficacy. Specifically, investigating the long-term impact of mindfulness-based interventions on eating behaviors and academic outcomes through extended and more frequent follow-ups can contribute to the refinement of intervention strategies. Finally, as orthorexic attitudes [65] and mindful eating [66] among students may be prone to cultural differences, the present findings need to be replicated and confirmed in future research in other countries and cultural backgrounds.

### Study limitations

The study at hand encountered inherent constraints, notably the cross-sectional design, which necessitates careful consideration when interpreting the findings as cause-and-effect relationship cannot be established. Additionally, the data were gathered via a questionnaire, which introduces a potential for bias associated with self-report measures and the subjective nature of responding to the items. The range of measurement tools and the limited number of studies delving into the intermediary function of psychological factors and mindful eating linking academic self-efficacy and orthorexia restricted our discussion. As a result, our discussion was rooted in hypotheses. The sample consisted only of female students, and thus findings may not be generalizable to males. There is a potential for residual confounding bias because not all factors linked to academic self-efficacy were considered into this study (such as premenstrual syndrome [67], dietary intake behaviors [68], comorbid mental health issues [69], profession (athletes, models) [70], having polycystic ovary syndrome [71], academic performance based on the cumulative grade point average, and a history of repeating semesters/failure [72]). For instance, marital status and religion were omitted; considering Lebanon’s population makeup, Muslims and Christians may have distinct dietary practices. Additionally, participants were not screened for eating disorders prior to data collection. Conversely, the utilization of a random sampling technique to choose our participants enables the extrapolation of our findings to the broader Lebanese population.

## Conclusion

Our findings indicated that focusd eating and psychological distress acted as a mediator between ON and academic self-efficacy. Understanding the influence of these factors can offer educators and policymakers valuable insights as they work to create comprehensive strategies that nurture and facilitate students’ academic journeys. These outcomes establish a foundation for future investigations into the interplay of mindful eating behavior, psychopathology, orthorexia, and academic self-efficacy across diverse populations.

## Data Availability

All data generated or analyzed during this study are not publicly available due the restrictions from the ethics committee, but are available upon a reasonable request from the corresponding author.
